# Mechanisms driving heme insertion into apo-sGC during its maturation in cells

**DOI:** 10.1186/2050-6511-14-S1-O18

**Published:** 2013-08-29

**Authors:** Dennis Stuehr, Arnab Ghosh, Anindya Sarkar, Yue Dai

**Affiliations:** 1 Department of Pathobiology, Lerner Research Institute, Cleveland Clinic, Cleveland Ohio, USA

## Background

During maturation of soluble guanylyl cyclase (sGC), heme insertion into the beta subunit is essential because it enables sGC to recognize nitric oxide (NO) and transduce its biological effects. We used a mammalian cell culture approach and followed heme insertion into both transiently- and endogenously-expressed apo-sGC beta. Although sGC is often associated with heat shock protein 90 (hsp90) in cells, the implications are unclear. Experiments that used pharmacological hsp90 inhibitors, an ATP-ase inactive hsp90 mutant, and heme-dependent or heme-independent sGC activators revealed that heme insertion into apo-sGC requires hsp90 [1].

## Results

Our findings suggest a model where apo-sGC beta may directly complex with hsp90, which then drives heme insertion into apo-sGC beta through its inherent ATPase activity. In follow-up studies we are using purified proteins and domains and fluorescence and hydrogen-deuterium exchange methods to characterize the apo-sGC interaction with hsp90 at the molecular level. Results indicate that apo-sGC beta binds to the hsp90 middle domain, and the binding interaction involves structural regions within the heme and PAS domains of sGC.

## Conclusion

Together, our work is shedding light on the sGC maturation process, and is revealing new ways that sGC activity could be impacted or controlled in cells.
